# Navigating Complexity in Mandibular Condyle Aplasia and Temporomandibular Joint Ankylosis in a Five-Year-Old Child: A Case Report

**DOI:** 10.7759/cureus.59615

**Published:** 2024-05-03

**Authors:** Keta Vagha, Sri Sita Naga Sai Priya K, Chaitanya Kumar Javvaji, Ashish Varma, Nitin Bhola, Gaurav Dubey, Shashank Agrawal

**Affiliations:** 1 Pediatrics, Jawaharlal Nehru Medical College, Datta Meghe Institute of Higher Education and Research, Wardha, IND; 2 Oral and Maxillofacial Surgery, Sharad Pawar Dental College and Hospital, Datta Meghe Institute of Higher Education and Research, Wardha, IND; 3 Radiology, Jawaharlal Nehru Medical College, Datta Meghe Institute of Higher Education and Research, Wardha, IND; 4 Medical School, Jawaharlal Nehru Medical College, Datta Meghe Institute of Higher Education and Research, Wardha, IND

**Keywords:** costochondral graft, craniofacial anomalies, facial asymmetry, temporomandibular joint ankylosis, mandibular condyle aplasia

## Abstract

Mandibular condyle aplasia and temporomandibular joint (TMJ) ankylosis represent complex challenges in diagnosis and management, affecting jaw function and facial aesthetics. This case report presents a five-year-old female child with a right-sided small jaw and facial asymmetry due to left-sided TMJ ankylosis. The coexistence of mandibular condyle aplasia and TMJ ankylosis underscores the need for comprehensive evaluation and tailored treatment approaches. Syndromic associations, such as Goldenhar syndrome and Treacher Collins syndrome, further complicate diagnosis and management. Surgical intervention involving left-side gap arthroplasty and reconstruction using a costochondral graft/temporalis fascia was performed under general anesthesia. However, postoperative complications, including decreased mouth opening and left-sided lower motor neuron facial palsy, necessitated further surgical debridement and drainage of an abscess. The case emphasizes the importance of a multidisciplinary approach in addressing complex craniofacial anomalies, with treatment strategies such as bone grafting and tailored surgical interventions offering promising outcomes. Understanding the multifaceted etiology of mandibular condyle aplasia and TMJ ankylosis is crucial for optimal management, highlighting the collaborative efforts required for achieving favorable patient outcomes.

## Introduction

Temporomandibular joint (TMJ) ankylosis, marked by the abnormal fusion between the condyle and the glenoid fossa, constitutes a prevalent etiology of acquired mandibular deformities, affecting individuals spanning various age groups, including both pediatric and adult populations [1]. The resultant restricted mouth opening and compromised functional movements associated with TMJ ankylosis contribute to mandibular growth impairment, often leading to observable manifestations such as retrognathism and facial asymmetry [2]. While unilateral TMJ ankylosis engenders asymmetrical mandibular growth, potentially accompanied by occlusal canting due to compensatory over-eruption of maxillary teeth on the contralateral side, bilateral involvement typically precipitates more pronounced deformities, including significant retrognathism, increased overjet, dental crowding, and the potential development of obstructive sleep apnea due to constriction of the oropharyngeal airway [3]. According to a study conducted by Gupta et al., the overall prevalence of TMJ ankylosis was reported as 0.46 per 1000 children in the population, demonstrating a female preponderance [4]. Hospital-based investigations have consistently shown that the most prevalent age groups presenting with TMJ ankylosis are 6-10 years and 11-20 years [5,6]. Trauma emerged as the most common etiological factor contributing to ankylosis [4].

Mandibular hypoplasia emerges as a consequential anomaly of the mandible, presenting as either congenital, developmental, or acquired in origin [7]. Congenital mandibular hypoplasia exhibits a diverse spectrum of clinical presentations, including both syndromic and nonsyndromic forms. Nonsyndromic variants encompass TMJ ankylosis, aglossia/macroglossia, and rare craniofacial clefts. In contrast, syndromic etiologies encompass a broader range of conditions, such as oculo-auriculo-vertebral syndrome, hemifacial macrosomia, bilateral facial macrosomia, Treacher Collins syndrome, and Goldenhar syndrome [8]. The management of mandibular condyle aplasia and TMJ ankylosis necessitates a comprehensive therapeutic approach, integrating surgical reconstruction, orthodontic interventions, and adjunctive therapies to optimize functional outcomes and mitigate associated morbidities. Surgical strategies encompass a spectrum of techniques, from condylar reconstruction and distraction osteogenesis to TMJ arthroplasty or total joint replacement, tailored to the individual patient's anatomical variations and clinical needs [9].

Despite advancements in surgical methodologies and adjunctive treatments, the management of mandibular condyle aplasia and TMJ ankylosis remains challenging, particularly in cases presenting with concurrent anomalies or secondary deformities. In this context, we present a compelling case report detailing the intricate management of mandibular condyle aplasia and TMJ ankylosis in a young patient, underscoring the complexities encountered and the tailored therapeutic approach implemented to address this rare clinical entity.

## Case presentation

**Figure 1:**
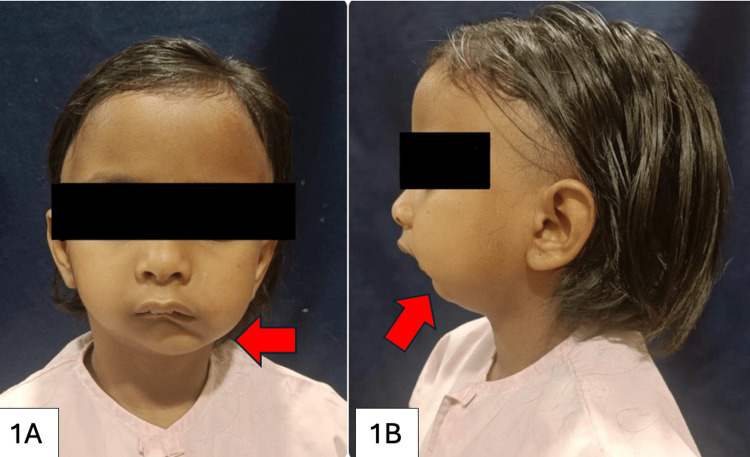
1A: Frontal view of the face showing facial asymmetry (red arrow). 1B: Lateral view of the face showing a retruded chin (red arrow).

A five-year-old female child presented at our tertiary care hospital in central India, with her father as the informant, complaining of facial asymmetry and a small jaw on her right side, accompanied by an inability to open her mouth since birth. As the child progressed, her grandmother noticed a relatively smaller lower jaw compared to the upper half of her face. The child encountered speech difficulties, manifesting in soft, slurred words, and encountered challenges with eating and biting small food pieces due to restricted mouth opening. There was no history of trauma reported. Upon examination, slight facial asymmetry was observed, along with mandibular deviation towards the left side upon opening, without mandibular retrognathism. There were no evident facial dysmorphism or anomalies in the ears. Additionally, elongation of the face on the unaffected side was noted, as depicted in Figure 1A, 1B.

**Figure 2:**
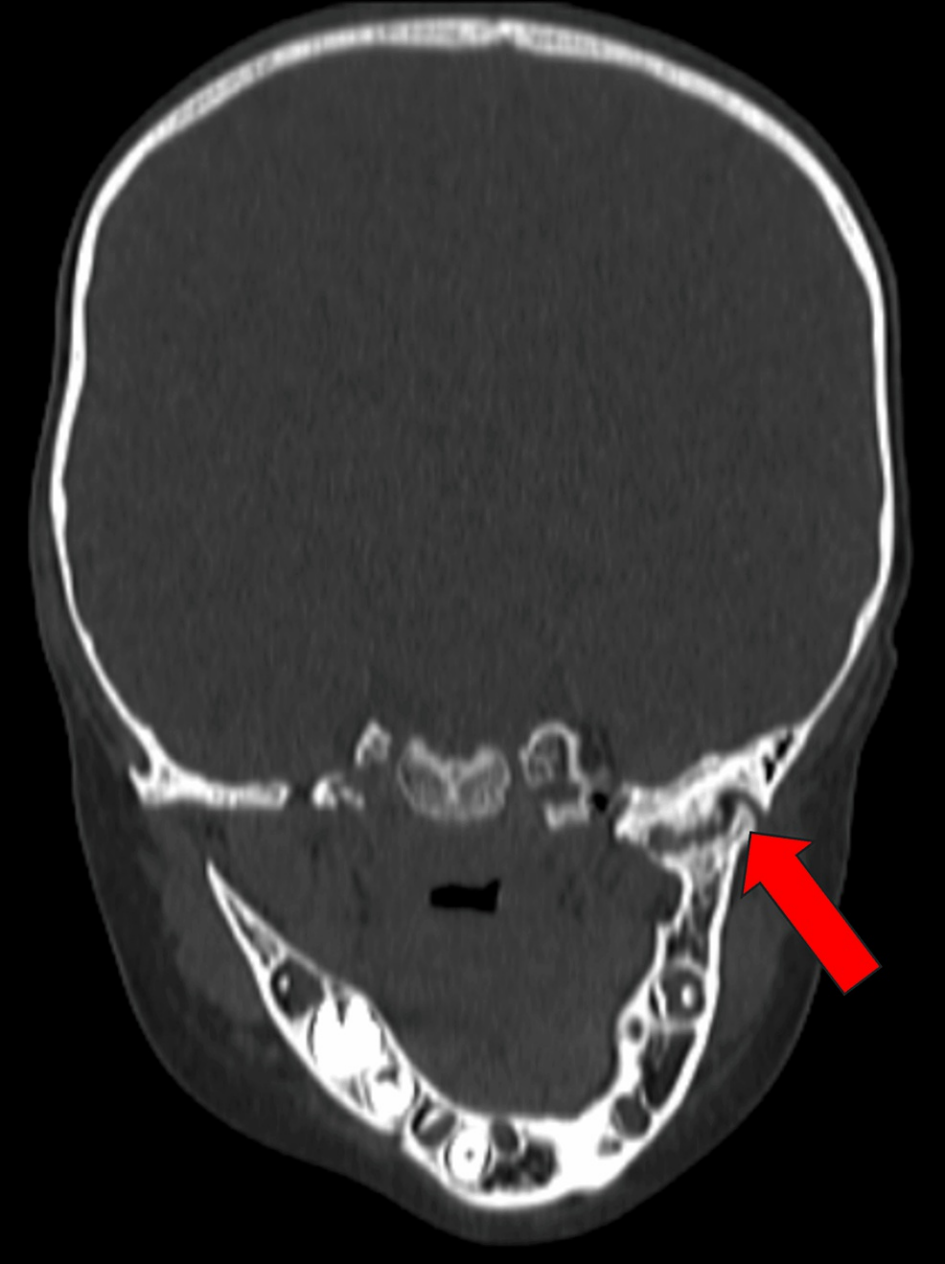
Coronal CT bone window of TMJ showing TMJ ankylosis (red arrow). CT: computed tomography; TMJ: temporomandibular joint

The TMJ examination revealed restricted mouth opening and mandibular deviation towards the affected side during opening. The potential diagnoses considered included TMJ ankylosis, hypertrophy of the coronoid process, fibrosis of the temporalis muscle, mandibular hypoplasia, and myositis ossificans. A computed tomography (CT) scan of the face was recommended to confirm the diagnosis. The child was diagnosed with left-sided TMJ ankylosis based on the CT of the face (Figure 2).

**Figure 3:**
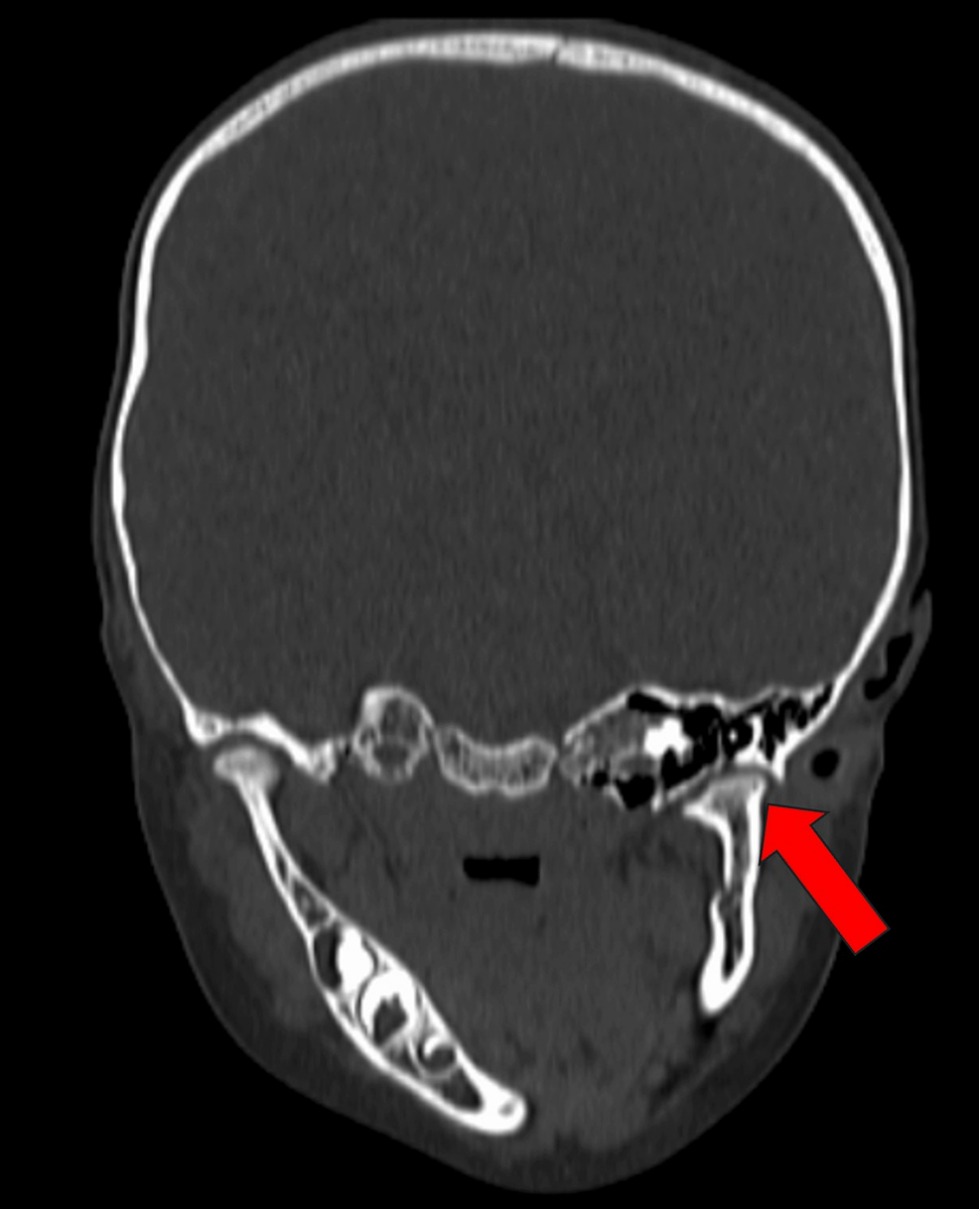
Coronal CT bone window of TMJ showing left mandibular hypoplasia with tilt of the mandible (red arrow). CT: computed tomography; TMJ: temporomandibular joint

The CT scan report indicates a relatively small-sized mandibular condyle with an underdeveloped ramus of the left hemimandible, resulting in ipsilateral deviation of the chin and mandible to the left side, accompanied by mandibular retraction (Figure 3). Crowding of teeth was observed anteriorly in both the upper and lower jaw, with protrusion of maxillary teeth. Additionally, irregularity of TMJ was noted on the left side, along with reduced joint space.

The child underwent surgical intervention, including left-side gap arthroplasty and reconstruction using a costochondral graft/temporalis fascia under general anesthesia. An ankylotic mass surrounding the left TMJ joint was excised to create a gap, followed by gap arthroplasty. However, postoperatively, the patient exhibited decreased mouth opening on the sixth day, which, upon further examination, was attributed to pus collection over the left parotid region, leading to surgical debridement and drainage of the abscess. Following the surgical procedure, the patient developed lower motor neuron facial nerve palsy on the left side, resulting in ipsilateral paralysis affecting the left half of the face (Video 1).

**Video 1:** Video demonstrating lower motor neuron facial nerve palsy on the left side of the child.

**Figure 4:**
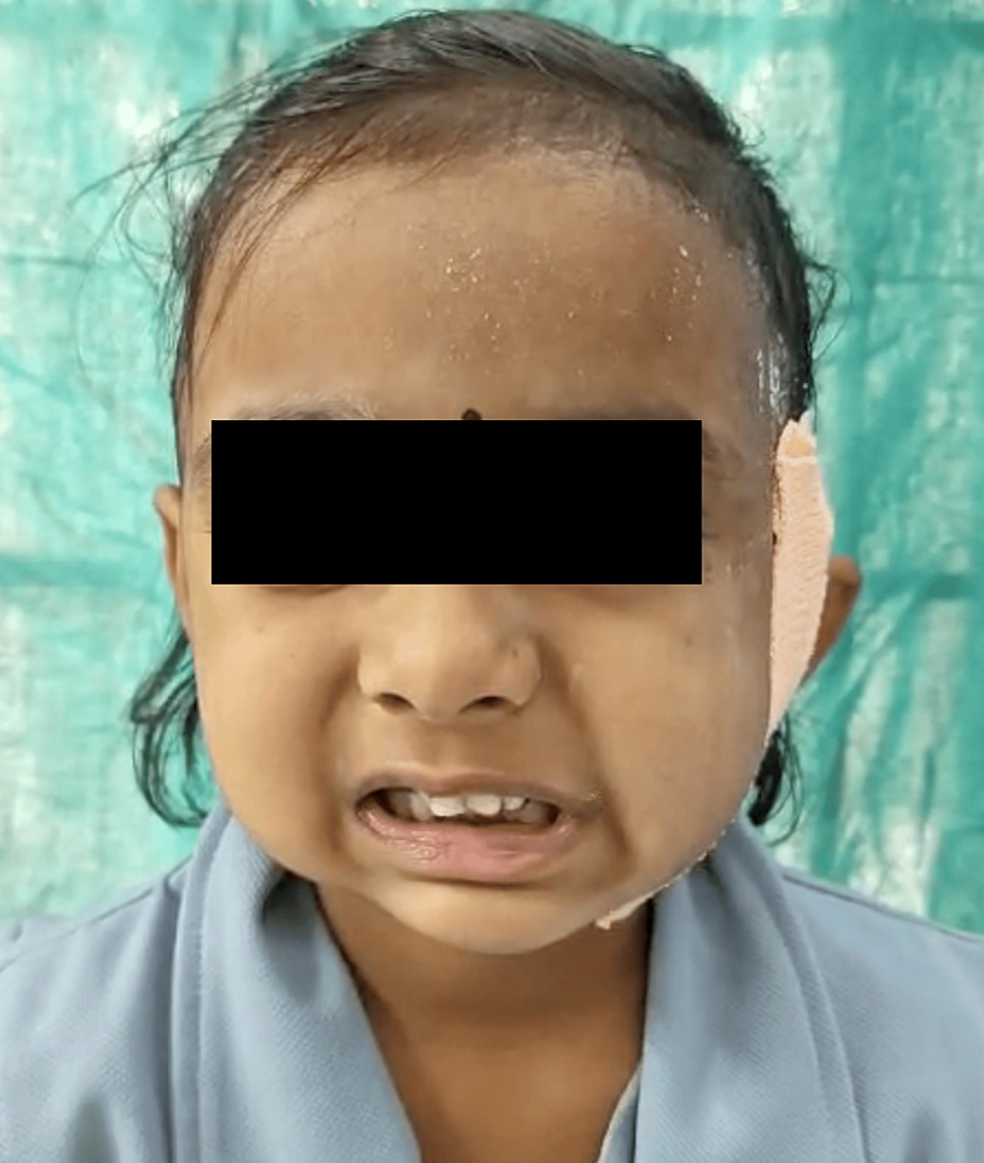
Postoperative image showing deviation of the angle of the mouth towards the opposite side.

The clinical manifestation of Bell's phenomenon and the deviation of the mouth's angle towards the opposing side were observed (Figure 4).

Consequently, the patient was commenced on a course of prednisolone for seven days, with a subsequent plan for tapering over an additional seven-day period. Despite our attempts to schedule regular follow-up appointments, the patient's attendance remained inconsistent, leading to their eventual loss of follow-up.

## Discussion

TMJ stands out as one of the most intricate joints in the human body, with its development commencing as early as the eighth week of gestation [10]. Disruptions in mandibular condyle growth during the first trimester can result in hypoplasia or aplasia, with condylar aplasia denoting a failure of development that can manifest unilaterally or bilaterally [10]. Notably, condylar aplasia, unaccompanied by additional facial malformations, is exceedingly uncommon [11]. Its incidence is estimated to be approximately one in 5,600 births, with congenital cases representing a mere 20%, as noted by Converse and Tideman alongside Doddridge [12]. TMJ ankylosis refers to the bony or fibrous fusion of the joint components, resulting in restricted mouth opening. This restriction can lead to challenges in chewing, speaking, and maintaining oral hygiene. Additionally, it may affect the symmetry of the facial skeleton, mainly if it occurs during a patient's growth phase [13].

The study discusses a case involving pediatric unilateral TMJ ankylosis and micrognathia. In this instance, TMJ ankylosis was present from birth, resulting in facial asymmetry. Alongside normal growth, the patient experienced significant functional and aesthetic irregularities, potentially negatively impacting their social and psychological development [14]. The case presented sheds light on the intricacies of TMJ pathology and its impact on mandibular development. TMJ ankylosis, characterized by fusion or immobility of the joint, can lead to significant functional limitations and facial asymmetry [15]. In our case, the child exhibited symptoms consistent with left-sided TMJ ankylosis, including restricted mouth opening and mandibular deviation towards the affected side.

Mandibular development is a multifaceted process that begins early in gestation, involving the migration of neural crest cells and subsequent formation of the first branchial arch. Disruptions in this process, such as insufficient migration of neural crest cells, can result in congenital anomalies such as mandibular aplasia or hypoplasia [16]. Factors contributing to mandibular abnormalities include genetic predisposition, environmental influences, and developmental anomalies. The categorization of congenital condylar defects serves as a framework for tailoring treatment strategies according to the underlying etiology. The differentiation between structurally altered and developmentally impaired causes of mandibular abnormalities informs surgical interventions and prognostic assessments. Syndromic associations like Goldenhar syndrome and Treacher Collins syndrome may involve additional craniofacial anomalies, complicating treatment planning and management processes.

Among the three types of mandibular hypoplasias, congenital mandibular hypoplasia arises from inadequate neural crest cell migration into the first branchial arch, leading to bilateral deformity. Developmental hypoplasias, characterized by class II malocclusion, have an unknown etiology. Acquired hypoplasias, encompassing oncologic defects, radiation damage, trauma, and hemifacial atrophy, are acquired later in life. In our case, the external appearance suggested right-sided mandibular hypoplasia; however, the associated left-sided TMJ ankylosis caused the jaw to be pulled towards the left side, creating the illusion of hypoplasia on the right side. This observation underscores the importance of comprehensive clinical assessment and radiographic evaluation in determining the true nature of mandibular abnormalities [17].

The initial advancements in surgical treatment methods for TMJ affected by ankylosis date back to 1851. During the decade spanning from 1850 to 1860, procedures such as condylectomy and arthroplasty were conducted, involving the creation of a new joint cavity and utilizing a myofascial flap [18]. Surgical management of TMJ ankylosis aims to restore joint function, alleviate symptoms, and address associated facial asymmetry. Gap arthroplasty and reconstruction with costochondral grafts are commonly employed techniques, offering favorable outcomes, particularly in pediatric patients [19]. Early intervention and aggressive postoperative rehabilitation are pivotal in preventing recurrence and optimizing long-term outcomes.

Engaging in early postoperative exercise, active physiotherapy, and consistent follow-up are crucial to prevent postoperative shrinkage and adhesions in cases of TMJ ankylosis. Success in maintaining postoperative mouth opening largely depends on the patient's motivation to undergo active mouth opening training and their ability to tolerate discomfort [20]. The case underscores the complexity of TMJ pathology and its implications for mandibular development. A multidisciplinary approach involving oral and maxillofacial surgeons, orthodontists, and physiotherapists is essential for comprehensive management. Advancements in surgical techniques and adjunctive therapies continue to improve outcomes and quality of life for patients with TMJ disorders.

## Conclusions

This case report underscores the complex challenges of mandibular condyle aplasia and TMJ ankylosis in pediatric patients. Surgical intervention with gap arthroplasty and costochondral graft reconstruction offers promising functional and aesthetic restoration outcomes. However, postoperative complications such as decreased mouth opening and facial nerve palsy require careful management. Syndromic associations further complicate diagnosis and treatment planning. This case highlights the necessity for a multidisciplinary approach and individualized care to address these complex craniofacial anomalies and optimize patient outcomes.
